# Toll-Like Receptor 9 Is Required for Opioid-Induced Microglia Apoptosis

**DOI:** 10.1371/journal.pone.0018190

**Published:** 2011-04-29

**Authors:** Lei He, Hui Li, Lin Chen, Junying Miao, Yulin Jiang, Yi Zhang, Zuoxiang Xiao, Gregory Hanley, Yi Li, Xiumei Zhang, Gene LeSage, Ying Peng, Deling Yin

**Affiliations:** 1 Department of Neurology, Sun Yat-sen Memorial Hospital, Sun Yat-sen University, Guangzhou, People's Republic of China; 2 Department of Internal Medicine, College of Medicine, East Tennessee State University, Johnson City, Tennessee, United States of America; 3 Department of Pharmacology, Shandong University School of Medicine, Jinan, People's Republic of China; 4 Institute of Developmental Biology, Shandong University School of Life Science, Jinan, People's Republic of China; 5 Department of Chemistry, East Tennessee State University, Johnson City, Tennessee, United States of America; 6 Cancer and Inflammation Program, Center for Cancer Research, National Cancer Institute at Frederick, Maryland, United States of America; 7 Division of Laboratory Animal Resources, College of Medicine, East Tennessee State University, Johnson City, Tennessee, United States of America; National Institute of Allergy and Infectious Diseases - Rocky Mountain Laboratories, United States of America

## Abstract

Opioids have been widely applied in clinics as one of the most potent pain relievers for centuries, but their abuse has deleterious physiological effects beyond addiction. However, the underlying mechanism by which microglia in response to opioids remains largely unknown. Here we show that morphine induces the expression of Toll-like receptor 9 (TLR9), a key mediator of innate immunity and inflammation. Interestingly, TLR9 deficiency significantly inhibited morphine-induced apoptosis in microglia. Similar results were obtained when endogenous TLR9 expression was suppressed by the TLR9 inhibitor CpGODN. Inhibition of p38 MAPK by its specific inhibitor SB203580 attenuated morphine-induced microglia apoptosis in wild type microglia. Morphine caused a dramatic decrease in Bcl-2 level but increase in Bax level in wild type microglia, but not in TLR9 deficient microglia. In addition, morphine treatment failed to induce an increased levels of phosphorylated p38 MAPK and MAP kinase kinase 3/6 (MKK3/6), the upstream MAPK kinase of p38 MAPK, in either TLR9 deficient or µ-opioid receptor (µOR) deficient primary microglia, suggesting an involvement of MAPK and µOR in morphine-mediated TLR9 signaling. Moreover, morphine-induced TLR9 expression and microglia apoptosis appears to require μOR. Collectively, these results reveal that opioids prime microglia to undergo apoptosis through TLR9 and µOR as well. Taken together, our data suggest that inhibition of TLR9 and/or blockage of µOR is capable of preventing opioid-induced brain damage.

## Introduction

Opioids have been used as potent analgesics for centuries; however, their abuse has deleterious physiological effects beyond addiction [1]–[3]- for example, they can somehow alter the functions of the immune and nervous systems [1], [4], [5]. Because opioid addicts are susceptible to certain infections, opioids such as morphine, have been suspected to suppress the immune response. This was supported by findings that various immune-competent cells express opioid receptors and undergo apoptosis when treated with opioid alkaloids [1]–[3], [6]. Our previous studies revealed that chronic morphine exposure, such as occurs in drug abuse, promotes apoptosis both *in vitro* and *in vivo* [1], [3], [7]–[9]. In the central nervous system (CNS), we and others have previously reported that the importance of opioid-induced apoptosis in neurons [7], [10]. Opioid receptors play critical roles in the processes of opioid-induced effects. All three opioid receptor types, μ, δ, and κ have been identified on microglia [4], [11].

Microglia are key players of the immune response in the CNS and represent the resident immune host defense and are considered the major immune inflammatory cells of the CNS [12], [13]. We and others have shown that morphine treatment-induced microglia apoptosis could be blocked by naltrexone or naloxone, specific opioid receptor antagonists, indicating a pivotal role of opioid receptors in this process [4], [14]. Our recent studies found that a deficiency of Toll-like receptor 2 (TLR2) significantly inhibits morphine-induced apoptosis in primary neurons [15].

TLRs are well known for recognition of pathogens in the innate immune system aimed at defending the survival of the host [16]–[18]. To date, the TLR family includes a total of 13 receptors that are responsible for the recognition of highly conserved structural motifs that are essential for pathogen survival and are conserved across broad subclasses of microorganism [17], [19], [20]. Each TLR family member, with the exception of TLR3, signals through the MyD88 dependent pathway [17]. TLRs and their functions have been established in immune cells [17], [18]. TLR3, TLR7, and TLR9 are distinct from other TLRs in that they are not expressed on the plasma membrane [19], [21]. TLR9 was identified as a key immune receptor in TLRs family that can recognize bacterial DNA as well as oligodeoxynucleotides (ODN) containing the CpG motifs responsible for the activating capacity of bacterial DNA (CpG s-ODN) [21], [22]. Activation of TLR9 signaling triggers activation of proapoptotic signals, and causes cell apoptosis in various systems [19], [21]. Growing evidence suggest that TLRs, including TLR9, expressed by microglial cells are critical in identifying and generating innate immune responses against bacterial and viral pathogens in the CNS [23]–[25]. Recently, it has been shown that TLR9, but not TLR2 or TLR4, plays a role in morphine inhibition of S.pneumoniae-induced NF-κB activity in the early stage of infection [26]. We have recently shown that morphine through potent stimulus of TLR9 caused altered host resistance against Mycobacterium tuberculosis in the lung of mouse model [19]. However, the role of TLR9 signaling on opioid-mediated apoptosis remains unknown.

One of three major subfamilies of mitogen-activated protein kinases (MAPKs) p38 MAPK plays a pro-apoptotic role [4], [27], [28]. Chronic morphine administration enhances the phosphorylation of p38 MAPK in dorsal root ganglion neurons [29]. Inhibition of p38 MAPK by p38 inhibitor SB203580 significantly attenuated tolerance to morphine analgesia [30]. P38 MAPK seems to sensitize cells to apoptosis by up-regulating Bax [31], [32], a pro-apoptotic member of the Bcl-2 family [31], [32]. The role of TLR9 in p38 MAPK and Bcl-2-mediated microglia apoptosis is not known yet. The objective of this study was to investigate the mechanisms by which opioids prime TLR9-mediated microglia apoptosis. Specifically we determined the involvement of µOR-mediated pro-apoptotic p38 MAPK and the upstream MAPK kinase (MKK3/6) of p38 MAPK, and Bcl-2 pathways. Here, we show that TLR9 is required for morphine-induced microglia apoptosis through p38 MAPK signaling pathway. Moreover, morphine promotes primary microglia apoptosis in a µ opioid receptor (µOR) dependent mechanism. Our studies thus provide an important insight into the mechanism of microglia apoptosis in response to opioids.

## Results

### Increased TLR9 expression in wild type microglia following morphine treatment

**Figure 1:**
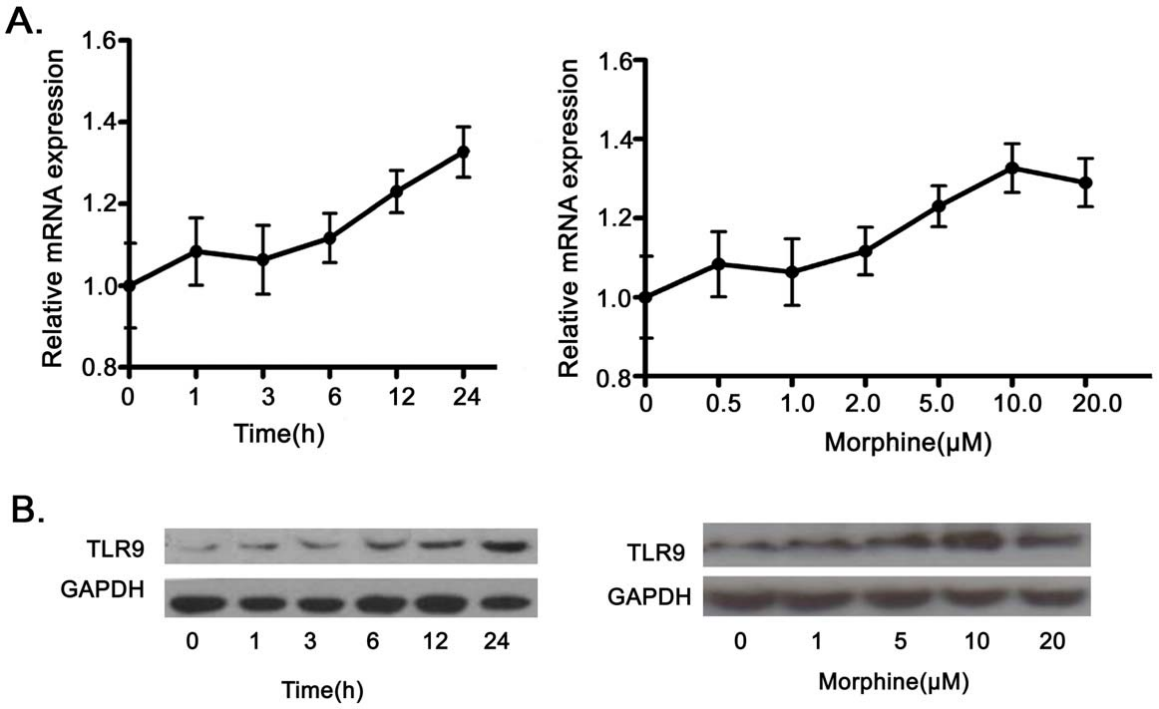
Morphine induces TLR9 expression in a dose and time dependent manner in wild type microglia. (A). Morphine induces the expression of TLR9 in mRNA level in a dose and time dependent. Mouse primary microglial cells were treated with 10 µM morphine for various time periods or different concentrations of morphine for 12 hr, respectively. The expression of TLR9 was determined by quantitative real time RT-PCR as described under Materials and Methods. Results represent mean ± SD of three independent experiments. (B). TLR9 expression in protein levels were determined by western blot. Cells were treated with 10 µM morphine for various time or various concentrations of morphine for 24 hr, respectively. Data are representative of three independent experiments.

Although recent studies have demonstrated that TLR9 is expressed on microglia, the molecular mechanisms by which opioids affect microglia function in unknown [4], [33]. To define the mechanisms, we treated primary mouse microglia from wild type mice with various concentrations of morphine for different time periods and examined the expression of TLR9 by quantitative real time RT-PCR and Western blot analysis. TLR9 expression in mRNA level was significantly increased following morphine treatment (Fig. 1A). Morphine-induced TLR9 expression was also detected at the protein level (Fig. 1B). The results demonstrated that morphine induces TLR9 expression in primary microglia in a dose and time dependent manner.

### TLR9 deficiency prevents morphine-induced microglia apoptosis

**Figure 2:**
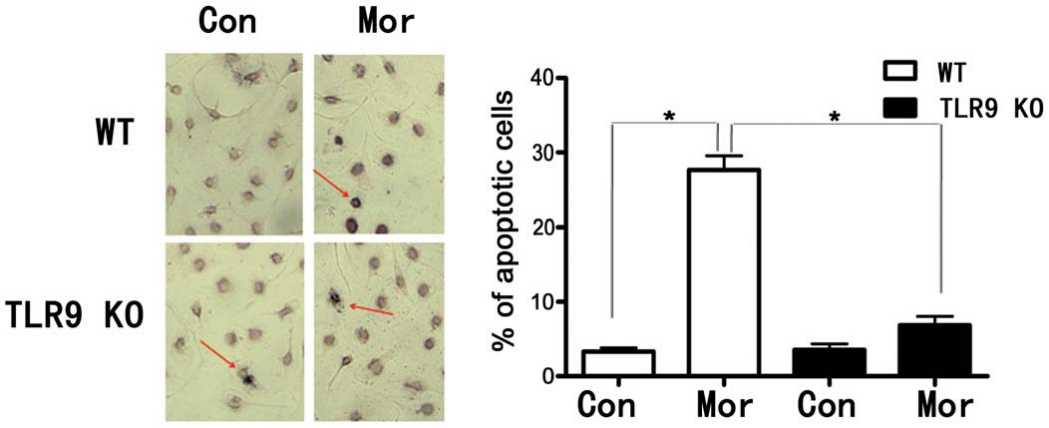
A deficiency of TLR9 is resistant to morphine-induced microglia apoptosis. Wild type (WT) and TLR9 deficient (TLR9 KO) microglia were treated with or without 10 µM morphine for 24 hr. Apoptotic cells (dark brown color cells) were determined by TUNEL assay. Photographs of representative TUNEL-stained cells are shown at the top. Magnification 200×. The bar graph shows the percentage of apoptotic cells. Results represent mean ± SD from three independent experiments. * *p*<0.01 compared with indicated groups.

We have recently reported that morphine promotes apoptosis in micgroglia [4]. To investigate whether TLR9 plays a role in opioid-induced microglia apoptosis, we treated primary mouse microglia from TLR9 knockout mice and wild type mice with 10 µM morphine for 24 h and evaluated cell apoptosis by TUNEL assay [4], [15], [19]. As shown in Fig. 2, a significant number of cells in the wild type microglia following morphine treatment were undergoing apoptosis, whereas only a few apoptotic cells were detected in the TLR9 deficient microglia after morphine treatment. Therefore, our results suggest TLR9 is required in opioid-mediated apoptosis in primary microglia.

### Blockade of TLR9 attenuates morphine-induced microglia apoptosis

**Figure 3:**
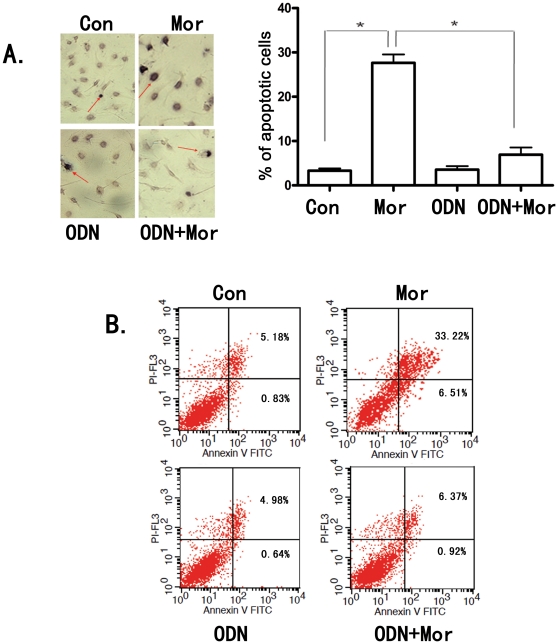
Inhibtion of TLR9 by CpGODN2088 blocked morphine-induced apoptosis in wild type microglia. WT microglial cells were exposed to 5 µM CpGODN2088 (ODN) for 1 hr and then treated with morphine at 10 µM for 24 hr. (A). Apoptotic cells were determined by TUNEL assay as in Fig. 2. Representative light microscopic images showed TUNEL-positive microglia (red arrow head) Results represent mean ± SD of three independent experiments. * *p*<0.01 compared with indicated groups. (B). Cell apoptosis was also assayed by flow cytometry after staining with annexin V and propidium iodide as described under “Materials and Methods”. These results are representative of three independent experiments.

Because TLR9 deficiency inhibited morphine-induced apoptosis, we postulated that TLR9 blockade might protect wild type microglia following morphine treatment. We pretreated wild type primary microglia cells for 1 hr with the TLR9 inhibitor CpGODN2088 [34] or vehicle, and then treated with morphine for 24 hr. Apoptotic cells were analyzed either by TUNEL assay (Fig. 3A) or by flow cytometric analysis (Fig. 3B). We showed that co-exposure of wild type microglia to CpGODN2088 and morphine resulted in a significant decrease in apoptosis compared to cells treated with morphine alone (Fig. 3), suggesting that this approach may be useful clinically.

### Inhibition of p38 MAPK diminishes morphine-induced microglia apoptosis

**Figure 4:**
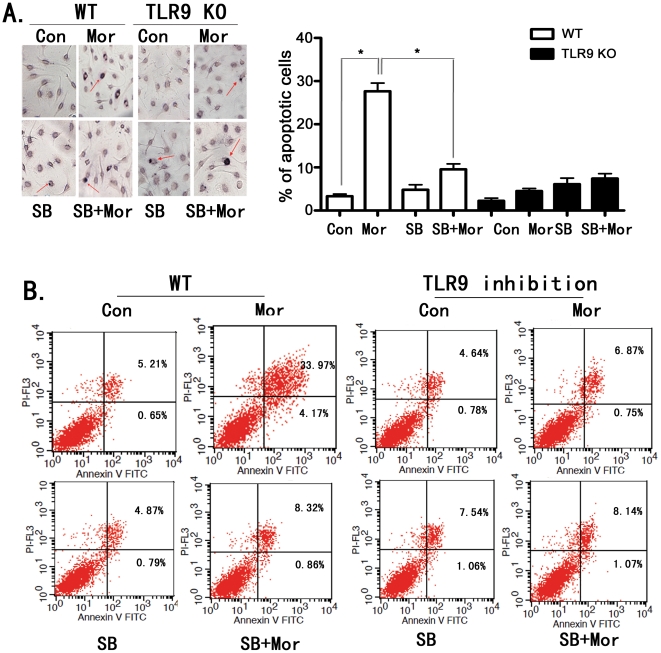
Inhibition of p38 MAPK by SB203580 attenuates morphine-induced microglia apoptosis. WT microglial cells and TLR9 deficient microglial cells were pretreated with 10 µM SB203580 (SB) for 1 hr and then exposed to morphine at 10 µM for 24 hr. Apoptotic cells were determined as in Fig. 3. (A). Representative light microscopic images showed TUNEL-positive microglia (red arrow head). Magnification 200×. Results represent mean ± SD from three independent experiments. * *p*<0.01 compared with indicated groups. (B). Apoptotic cells were analyzed by flow cytometry. Three experiments were performed with similar results.

Opioids promote macrophage apoptosis through p38 MAPK phosphorylation [35]. To determine the role of p38 MAPK on TLR9-mediated microglia apoptosis induced by morphine exposure, we pretreated wild type and TLR9 deficient primary microglial cells with the specific p38 MAPK inhibitor SB 203580 [4] and then treated the cells in the presence or absence of morphine. We determined cell apoptosis by either TUNEL assay (Fig. 4A) or flow cytometry (Fig. 4B). As shown in Fig. 4, inhibition of p38 MAPK by SB203580 significantly attenuated morphine-induced apoptosis in wild type primary microglial cells. SB203580 did not alter the effects of morphine on TLR9 deficient microglial cells. SB203580 alone did not induce apoptosis in either wild type or TLR9 deficient microglial cells.

### Inhibition of p38 MAPK attenuates morphine-induced alteration of Bcl-2 and Bax expression

**Figure 5:**
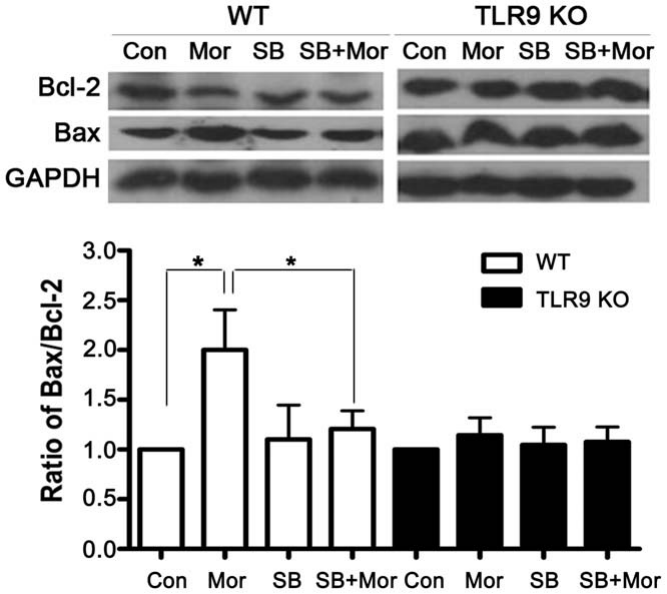
Blockade of p38 MAPK by SB203580 inhibits morphine-induced changes in Bcl-2 and Bax expression levels. WT microglia were exposed to SB203580 (SB) at 10 µM and then treated with morphine at 10 µM for 24 hr. The expression of Bax and Bcl-2 was examined by Western blot analysis. Representative results of the levels of Bcl-2 and Bax are shown of each pane. Mean values were derived from three independent experiments. * *p*<0.01 compared with indicated groups.

It has been reported that p38 MAPK modulates Bcl-2/Bax-mediated apoptosis in neuroblastoma cells [32], [36]. Our previous studies have reported that treatment of morphine with R37Ra protein significantly decreased the level of Bcl-2 and increased the level of Bax in the lung from wild type mice [19]. To examine whether p38 MAPK modulates Bcl-2 signaling following morphine treatment in TLR9-mediated signaling, we examined the expression of anti-apoptotic Bcl-2 and pro-apoptotic Bax by Western blot. Primary wild type microglia and TLR9 deficient microglial cells were treated with SB203580 for 1 hr and then treated with or without morphine for 24 hr. Morphine treatment significantly enhanced the expression of Bax but decreased Bcl-2 expression in wild type microglial cells (Fig. 5). In contrast, morphine did not alter the levels of Bcl-2 and Bax in the microglia from TLR9 knockout mice, suggesting that Bcl-2 family participates in TLR9-mediated microglia signaling following morphine treatment. Intriguingly, inhibition of p38 MAPK by SB203580 significantly diminished morphine-induced changes of Bcl-2 and Bax compared with the morphine treatment alone in wild type microglial cells, but not in TLR9 deficient microglial cells. SB203580 alone did not alter the level of Bax (Fig. 5). Thus, our data suggest that TLR9 plays a critical role in p38 MAPK-mediated Bcl-2 signaling in microglial cells following morphine treatment.

### Effect of TLR9 and μOR on the levels of phosphor-p38 MAPK and phosphor-MKK3/6 following morphine treatment

**Figure 6:**
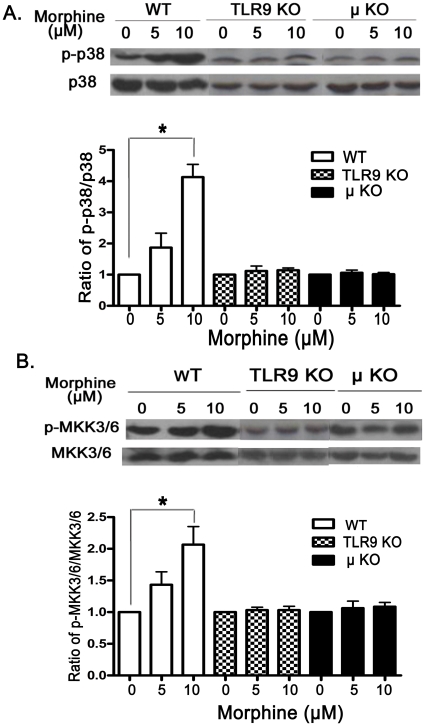
Effect of morphine on p38 MAPK and MEKK3/6 activation. WT, TLR9 KO, and µOR KO microglial cells were treated with morphine at 5 and 10 µM for 24 hr. (A) Total and phosphorylated p38 MAPK (p-p38 MAPK) levels were determined by Western blot. Mean values were derived from three independent experiments. * *p*<0.01 compared with indicated groups. (B) Total and phosphorylated MKK3/6 (p-MKK3/6) levels were examined by Western blot. Mean values were derived from three independent experiments. * *p*<0.01 compared with indicated groups.

Since inhibition of p38 MAPK could effectively block the morphine-induced apoptosis in wild type microglial cells (Fig. 4), its consequence on the level of phosphorylation of p38 MAPK was next determined in wild type and TLR9 deficient microglial cells with 10 µM morphine treatment for 24 hr. As shown in Fig. 6A, morphine significantly enhanced the level of phospho-p38 MAPK in wild type microglial cells. The increase of phosphor-p38 in wild type microglial cells was attenuated markedly in TLR9 deficient primary microglia. We have shown that morphine induces microglia apoptosis through opioid-receptors [4]. To examine the role of μOR, a key opioid receptor of opioids' actions, we determined the level of phospho-p38 MAPK in μOR deficient microglial cells following morphine treatment. We found that a deficiency of μOR in primary microglia strongly suppressed the level of P38 phosphorylation compared with wild type primary microglia following morphine treatment (Fig. 6A).

Next, we examine the of phosphorylation level of MKK3/6, the upstream MAPK kinase of p38 MAPK, in microglial cells with 10 µM morphine treatment for 24 hr. Morphine treatment resulted in a dramatic increase of MKK3/6 phosphorylation level in wild type primary microglia, but not in TLR9 or μOR deficient primary microglia (Fig. 6B). Taken together, these results suggest that p38 MAPK and MKK3/6 are involved in morphine-mediated microglia apoptosis through TLR9 and/or μOR.

### Morphine induces microglia apoptosis through μOR

**Figure 7:**
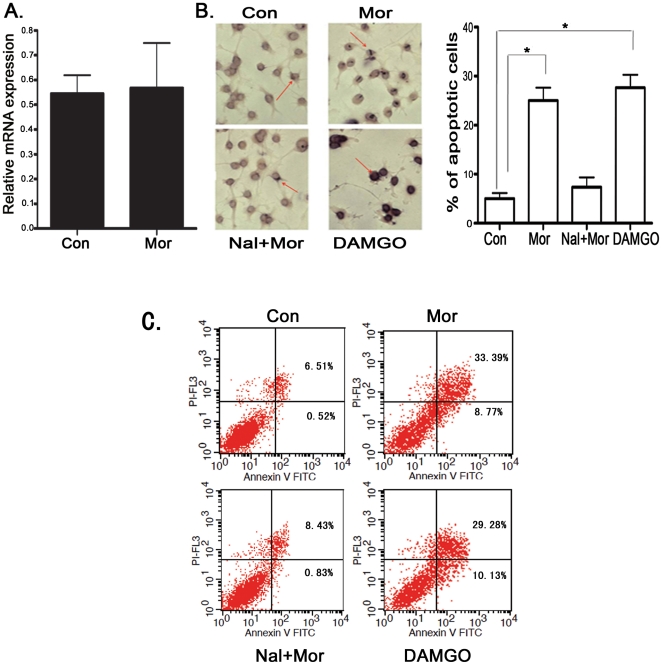
Morphine induces microglia apoptosis in a μOR dependent manner. (A) Morphine could not induce the expression of TLR9 in μOR KO microglia. μOR deficient microglia cells were treated morphine at 10 µM for 12 hr and the expression of TLR9 in mRNA levels determined by quantitative real time RT-PCR as in Fig. 1A. Wild type microglial cells were treated with 10 µM morphine with or without 20 µM naloxone pretreatment. Another group of cells were treated with 10 µM DAMGO alone for 24 hr. Apoptotic cells were examined as in Fig. 3. (B). Representative light microscopic images showed TUNEL-positive microglia (red arrow head). Magnification 200×. Results represent mean ± SD from three independent experiments. * *p*<0.01 compared with indicated groups. (C). Apoptotic cells were analyzed by flow cytometry. These results are representative of three independent experiments.

It has been established that several opioid receptor isoforms, including μOR, are expressed on microglia [11]. To test whether morphine-induced TLR9 is through μOR, we treated primary microglia from μOR knockout mice and from wild type mice with morphine at 10 µM and examined the expression of TLR9 by quantitative real time RT-PCR and immunohistochemistry. The expression of TLR9 was significantly induced by morphine in wild type microglia (data not shown), consistent with the findings of our results in Figure 1. Importantly, morphine could not alter the expression of TLR9 in μOR knockout microglia both in mRNA level (Fig. 7A) and protein level (data not shown). Taken together, our results demonstrated that morphine treatment significantly increases TLR9 expression through μOR.

We next investigated the role of opioid receptors, including μOR, in morphine-induced microglia apoptosis. Primary wild type microglial cells were exposed to morphine at 10 µM in the presence or absence of the opioid-receptor antagonist naloxone [4], [9], [37]. Apoptotic cells were examined either by TUNEL assay (Fig. 7B) or by flow cytometric analysis (Fig. 7C). We observed that pretreatment with naloxone dramatically blocked morphine-induced microglia apoptosis (Figs. 7B and 7C). Treatment of microglia with naloxone alone did not alter the percentage of apoptosis (data not shown). We next tested with a specific μOR agonist [D-Ala2, N-MePhe4, Gly-ol]-enkephalin (DAMGO). DAMGO significantly induced microglia apoptosis but no significant difference was observed when compared with the morphine treatment group (Figs. 7B and 7C). Morphine could not cause apoptosis in μOR deficient microglia (data not shown). Therefore, morphine-induced microglia apoptosis appears to require μOR.

## Discussion

The data presented herein revealed for the first time, to our best knowledge, a key role for microglia TLR9 in the induction of morphine-mediated apoptosis. Our studies demonstrate that morphine treatment modulates TLR9 signaling and results in microglia apoptosis. It is widely accepted that TLR9 signaling mainly activates inflammatory responses, including proinflammatory cytokines [18], [21], [22], [38]. Many studies focused attention on TLR9-mediated apoptosis in immune system [18], [21]. Recent studies have shown that apoptosis via TLR9 on immune cells may play a role in the pathogenesis of various autoimmune diseases [39]. In this study, we identify that deficiency of TLR9 activity in primary microglia blocked morphine-induced microglial apoptosis. Morphine concentration we used in this study is physiologically relevant and these levels can be achieved in the brains of patients given opioids [4], [14], [40], [41]. Our studies revealed that TLR9 signaling is activated by opioids in microglia without the presence of immune cells. We discovered that the absence of TLR9 blocked microglia apoptosis induced by morphine treatment (Fig. 2). Importantly, these features in TLR9 deficient microglia were replicated in wild type microglia through the administration of the TLR9 inhibitor ODN2088 (Fig. 3). Thus, our results demonstrated that TLR9 is required in morphine-induced apoptosis in microglia. This provides a possible target for control of morphine caused neurotoxicity and brain damage.

MAPKs are a family of serine/threonine kinases that perform important functions as mediators of cellular responses to various extracellular stimuli, including cell survival and apoptosis [27], [28]. MAPKs consist of three major subfamilies in mammalian cells [27], [28], such as p38 MAPK. The activation of p38 kinase activity generally promotes apoptosis [27]. We showed that inhibition of p38 MAPK attenuated morphine-induced apoptosis in microglia (Fig. 4). It has been shown that morphine treatment increases the phosphorylation of p38 MAPK in neurons [29]. We found in this study that morphine induces microglia p38 MAPK activation in wild type microglia but not in either TLR9 deficient microglia or μOR deficient microglia (Fig. 6). In addition, we identified that MKK3/6 was also activated in wild type microglia, but not in TLR9 deficient microglia or μOR deficient microglia, following morphine treatment. The results suggest that morphine's pro-apoptotic effects observed in microglia are due part to the activated MKK3/6-p38 MAPK signaling.

Recently, it has been shown that p38 is necessary for Bcl-2-induced inhibition of apoptosis in fibroblasts [42]. Previous studies observed that morphine induces lymphocyte apoptosis through mechanisms associated with a decrease in anti-apoptotic protein Bcl-2 expression and an enhancement in that of pro-apoptotic protein Bax [43], [44]. The role of Bcl-2 and Bax in p38 MAPK-mediated microglia apoptosis was investigated in our studies. We found that morphine significantly decreased the levels of Bcl-2 and increased the levels of Bax in the microglia from wild type mice. Interestingly, inhibition of p38 MAPK attenuated morphine-induced the alterations of Bcl-2 and Bax expression (Fig. 5). However, morphine did not alter the levels of Bcl-2 and Bax in the microglia from TLR9 knockout mice. Taken together, our studies demonstrate that Bcl-2 and Bax participate in TLR9-mediated p38 MAPK signaling in morphine-induced apoptosis in microglia.

Previous *in vitro* studies have demonstrated that morphine and DAMGO, a specific μ-opioid receptor agonist, induce apoptosis in lymphocytes [44]. Our results showed that morphine induces microglia apoptosis through a μOR dependent mechanism (Fig. 7). To discriminate between the eventual effect of morphine on TLR9 and μOR, we have made a novel observation that morphine could not induce TLR9 expression in primary μOR deficient microglia in both mRNA and protein levels. Therefore, the regulation of TLR9 by morphine described here is μOR dependent. However, it is unclear how up-regulation of TLR9 leads to apoptosis induced by either morphine or DAMGO. The mechanisms by which morphine as well as DAMGO leads the up-regulation of TLR9 to microglia apoptosis will be investigated in future studies. It has been recently reported that opioids induce glial action not though classical opioid receptors, but rather through non-stereoselective activation of TLR4 [45]. However, the role of non- stereoselective TLR4-mediated signaling remains under investigation [45]. To our best knowledge, we provided the fist evidence that morphine induces TLR9 expression through a classical μOR dependent mechanism. Further investigations should be performed to understand the mechanisms on TLR9 regulation by morphine via μOR.

## Materials and Methods

### Experimental animals

TLR9 knockout (TLR9 KO) mice were kindly provided by Dr. Shizuo Akira, Osaka University, Osaka, Japan via Dr. Dennis Klinman, National Cancer Institute, Frederick, MD [19]. Mu-opioid receptor KO mice were kindly provided by Dr. Srinivasa Raja, Johns Hopkins University School of Medicine, Baltimore, MD [8]. KO mice and wild type mice were maintained in the same room in the Division of Laboratory Animal Resources at East Tennessee State University (ETSU), a facility accredited by the Association for the Assessment and Accreditation of Laboratory Animal Care International (AAALAC). All aspects of the animal care and experimental protocols were approved by the ETSU Committee on Animal Care.

### Reagents

Morphine sulfate and naloxone were obtained from Sigma-Aldrich (St. Louis, MO). Antibodies, including total and phospho-p38 and MKK3/6, were purchased from Cell Signal Technology (Beverly, MA). TLR9 antibody was purchased from IMGENEX (San Diego, CA). The antibodies of GAPDH, Bax, and Bcl-2 were purchased from Santa Cruz Biotechnology (Santa Cruz, CA). P38 inhibitor SB203580 was obtained from Tocris Bioscience (Bristol, UK). TLR9 inhibitor ODN2088 was obtained from InvivoGen (San Diego, CA).

### Primary microglial cells culture

Mouse primary microglial cells were isolated from mixed glial cultures, as described in our previous studies [4], [46]. Briefly, primary mixed glial cultures were prepared from postnatal day 1–2 mice. Primary microglia were co-cultured with astrocytes in poly-D-lysine-coated 75-cm^2^ culture flasks in DMEM (Gibco BRL, Gaithersburg, MD) supplemented with 10% heat-inactivated fetal bovine serum (FBS) (Atlanta Biologicals, Lawrenceville, GA) and 1% Penicillin/Streptomycin. On days 10–14, microglial cells were harvested by shaking the cultures (180 rpm) and collecting the floating cells. These cells were seeded into plastic tissue culture flasks. After incubation at 37°C for 1 h, non-adherent cells were removed by replacing culture medium. The cells were grown in DMEM with 10% FBS and maintained at 37°C and 5% CO_2_. The purity of microglia was verified >95% by ricinus communis agglutinin-1 (RCA-1, a microglia marker) immunostaining.

### Western blot analysis

Western blot was performed as described previously in our published work [3], [46], [47]. Briefly, the cellular proteins were separated by SDS–polyacrylamide gel electrophoresis and transferred onto Hybond ECL membranes (Amersham Pharmacia, Piscataway, NJ). The membrane was then incubated at room temperature in a blocking solution composed of 5% skim milk powder dissolved in 1× TBS (10 mM Tris, pH 8.0, and 140 mM NaCl) for 1 h. The membrane was then incubated with the blocking solution containing the first antibody overnight at 4°C. After washing three times with TBS for 5 min, the blot was then incubated with a second antibody. The blot was again washed three times with TBS before being exposed to the SuperSignal West Dura Extented Duration substrate (Pierce Biotechnology, Rockford, IL). Band intensity was quantified by densitometric analyses using a densitometer.

### Quantification of apoptosis by TUNEL assay

The experimental cells were treated with different concentrations of morphine in the presence or absence of SB203580 for 24 h or ODN2088 for 1 h. Apoptotic cells were determined by terminal deoxynucleotidyl transferase biotin-d UTP nick end labeling (TUNEL) assay using a situ cell death detection kit (Roche Diagnostic, Indianpolis, IN) according to the manufacturer's instruction as described in our previous studies [19], [37], [47]. The percentage of apoptotic cells was calculated by counting approximately 500 cells.

### Flow cytometry

Apoptotic cells were examined by flow cytometry as described previously [1], [37], [48]. Briefly, cells (1×10^6^) were washed twice with cold PBS and then suspended cells in 200 µL of 1X binding buffer and 5 µL of fluorescein isothiocyanate (FITC)-labeled Annexin V (R&D Systems, Minneapolis, MN) for 20 minutes in the dark, and thereafter 300 µL of 1X binding buffer and 5 µL of propidium iodide (Sigma, St. Louis, MO) were added to each sample. After incubation at RT (25°C) for 10 min in the dark, the cells were analyzed for apoptosis on a FACScan flow cytometer with CellQuest software (Becton Dickinson). All data are representative of three independent experiments.

### Real-time quantitative RT-PCR

Total RNA was isolated from lungs by the VERSA GENE™ RNA Tissue Kit (Gentra SYSTEMS; Minnesota) and the real-time RT-PCR detection technique was performed as described in our previous publications [15], [19]. Briefly, first-strand cDNA was synthesized from 1 µg of total RNA using a Reaction Ready™ first strand cDNA synthesis kit (SABiosciences, Frederick, MD). cDNA was subjected to real-time quantitative PCR using Bio-Rad iCycler iQ Multicolor Real-Time PCR Detection System (Bio-Rad Life Science Research, Hercules, CA). TLR9 primer pairs were used: CCCTGGTGTGGAACATCAT (forward) and GTTGGACAGGTGGACGAAGT (reverse). PCR assay was performed in triplicate. The reaction conditions were: 50°C for 2 min, 95°C for 8 min 30 s, followed by 27 cycles of 95°C for 15 s, 60°C for 60 s, and 72°C for 30 s.

### Statistical analysis

Results were expressed as mean ± SD. Statistical significance was assessed by one-way analysis of variance (ANOVA) and the student's test. Prism 5.0 (GraphPad Software, San Diego, CA) was used for all calculations. A value of *p<*0.05 was considered statistically significant.
